# Use of PRECIS ratings in the National Institutes of Health (NIH) Health Care Systems Research Collaboratory

**DOI:** 10.1186/s13063-016-1158-y

**Published:** 2016-01-16

**Authors:** Karin E. Johnson, Gila Neta, Laura M. Dember, Gloria D. Coronado, Jerry Suls, David A. Chambers, Sean Rundell, David H. Smith, Benmei Liu, Stephen Taplin, Catherine M. Stoney, Margaret M. Farrell, Russell E. Glasgow

**Affiliations:** Group Health Research Institute, Seattle, USA; National Cancer Institute, Bethesda, MD USA; University of Pennsylvania Perelman School of Medicine, Philadelphia, USA; Kaiser Permanente Center for Health Research, Portland, OR USA; University of Washington, Seattle, WA USA; National Heart, Lung, and Blood Institute, National Institutes of Health, Bethesda, MD USA; Department of Family Medicine, University of Colorado School of Medicine, Denver, USA

## Abstract

**Background:**

The National Institutes of Health (NIH) Health Care Systems Research Collaboratory (NIH Collaboratory) seeks to produce generalizable knowledge about the conduct of pragmatic research in health systems. This analysis applied the PRECIS-2 pragmatic trial criteria to five NIH Collaboratory pragmatic trials to better understand 1) the pragmatic aspects of the design and implementation of treatments delivered in real world settings and 2) the usability of the PRECIS-2 criteria for assessing pragmatic features across studies and across time.

**Methods/Design:**

Using the PRECIS-2 criteria, five pragmatic trials were each rated by eight raters. For each trial, we reviewed the original grant application and a required progress report written at the end of a 1-year planning period that included changes to the protocol or implementation approach. We calculated median scores and interrater reliability for each PRECIS domain and for the overall trial at both time points, as well as the differences in scores between the two time points. We also reviewed the rater comments associated with the scores.

**Results:**

All five trials were rated to be more pragmatic than explanatory, with comments indicating that raters generally perceived them to closely mirror routine clinical care across multiple domains. The PRECIS-2 domains for which the trials were, on average, rated as most pragmatic on the 1 to 5 scale at the conclusion of the planning period included primary analysis (mean = 4.7 (range = 4.5 to 4.9)), recruitment (4.3 (3.6 to 4.8)), eligibility (4.1 (3.4 to 4.8)), setting (4.1 (4.0 to 4.4)), follow-up (4.1 (3.4 to 4.9)), and primary outcome (4.1 (3.5 to 4.9)). On average, the less pragmatic domains were organization (3.3 (2.6 to 4.4)), flexibility of intervention delivery (3.5 (2.1-4.5)), and flexibility of intervention adherence (3.8 (2.8-4.5)). Interrater agreement was modest but statistically significant for four trials (Gwet’s AC1 statistic range 0.23 to 0.40) and the intraclass correlation coefficient ranged from 0.05 to 0.31. Rating challenges included assigning a single score for domains that may relate to both patients and care settings (that is, eligibility or recruitment) and determining to what extent aspects of complex research interventions differ from usual care.

**Conclusions:**

These five trials in diverse healthcare settings were rated as highly pragmatic using the PRECIS-2 criteria. Applying the tool generated insightful discussion about real-world design decisions but also highlighted challenges using the tool. PRECIS-2 raters would benefit from additional guidance about how to rate the interwoven patient and practice-level considerations that arise in pragmatic trials.

**Trial registrations:**

Clinicaltrials.gov trial registrations: NCT02019225, NCT01742065, NCT02015455, NCT02113592, NCT02063867.

## Background

Pragmatic clinical trials are “primarily designed to determine the effects of an intervention under the usual conditions in which it will be applied”; they contrast with explanatory trials which “are primarily designed to determine the effects of an intervention under ideal circumstances” [1]. The United States (U.S.) National Institutes of Health (NIH) Healthcare Systems Research Collaboratory (the NIH Collaboratory) was established to advance large-scale pragmatic clinical trials through the conduct of pragmatic trial demonstration projects. These demonstration projects are being performed in large and diverse healthcare settings around the United States and allow exploration of best practices for appropriately designing pragmatic trials in addition to generating trial findings [2]. Each trial had a design phase for 1 year and a subsequent implementation phase if approved after a preliminary report. The NIH Collaboratory comprises the research teams that design and perform the individual trials; a coordinating center with expertise in design, biostatistics, bioethics, and electronic health data; and NIH scientists.

The purpose of this analysis was to measure the degree to which the NIH Collaboratory trials are pragmatic at both the design and implementation phases using a version of the Pragmatic Explanatory Continuum Indicator Summary framework (PRECIS-2) [3]. In addition, because all NIH Collaboratory trials begin with a yearlong planning phase to pilot test the intervention and evaluate feasibility of aspects such as outcome ascertainment methods and integration with workflow, we were able to study whether and how trial design changed from conceptualization to implementation. As a secondary goal, we sought to assess the usability of PRECIS-2 as a tool for assessing pragmatic features across studies and over time.

## Methods

### Ethics

The individual NIH Collaboratory Trials were approved by the relevant research ethics boards. This analysis did not require informed consent from raters nor ethical approval because the data sources were limited to the study protocols, not information about human subjects. In addition, all raters were co-authors of the paper rather than subjects of the research.

### Setting

The U.S. NIH is the largest medical research agency in the world. Through funding from the NIH, the NIH Collaboratory seeks “to strengthen the national capacity to implement cost-effective large-scale research studies that engage health care delivery organizations as research partners. The aim of the program is to provide a framework of implementation methods and best practices that will enable the participation of many health care systems in clinical research” [4]. The NIH Collaboratory funded five pragmatic clinical trials at both a planning and implementation phase in 2012 and 2013, respectively. These trials are described in Table 1 and include 1) Active Bathing to Eliminate (ABATE) Infection, 2) Lumbar Image Reporting with Epidemiology (LIRE), 3) Collaborative Care for Chronic Pain in Primary Care (PPACT), 4) Strategies and Opportunities to Stop Colorectal Cancer (STOP CRC), and 5) Time to Reduce Mortality in End-Stage Renal Disease (TiME). Although additional trials have been funded through the Collaboratory, they are not included in this analyses as they had not been awarded funding for the implementation phase until completion of the analyses.

**Table 1:** Summary of included projects

Project title	Research question	Setting	Patient population	Design	Example of changes during planning period
Active Bathing to Eliminate Infection (ABATE Infection)	Does routine daily bathing with antiseptic soap for all patients plus targeted use of a nasal antibiotic ointment for MRSA carriers reduce multidrug resistant organisms and bloodstream infections in general medical, surgical, and oncology inpatient units?	Hospital Corporation of America (HCA)	Inpatients in non-critical care units at 53 U.S. HCA hospitals. Units where chlorhexidine bathing or nasal decolonization is common were excluded.	Cluster randomized trial of hospitals to compare two quality improvement strategies to reduce multidrug resistant organisms and bloodstream infections in non-critical care units. The two strategies to be evaluated are:	No changes of note.
				Arm 1: Routine Care - Routine policy for showering/bathing	
				Arm 2: Decolonization - Use of chlorhexidine as routine soap for showering or bed bathing for all patients plus mupirocin × 5 days if MRSA+ by history, culture, or screen	
A Pragmatic Trial of Lumbar Image Reporting with Epidemiology (LIRE)	Does adding epidemiologic benchmark data to spine imaging reports decrease subsequent back-related healthcare utilization?	Primary care clinics within the Kaiser Permanente-Northern California, Group Health Cooperative, Mayo Clinic Health System, and Henry Ford Health System	Approximately 150,000 adults for whom a primary care provider has requested imaging of the lumbar spine	Cluster randomized trial comparing typical imaging reports to those that include benchmarks prevalence data of findings in patients without back pain.	Clinics with a single provider were excluded, making recruitment slightly more restricted.
Collaborative Care for Chronic Pain in Primary Care (PPACT)	Does an interdisciplinary team-based program sited in primary care help patients manage chronic pain?	Primary practices in three Kaiser Permanente regions	Approximately 1,000 patients prioritized by their providers who have nonmalignant chronic pain and who are on long-term opioid therapy.	Mixed-methods cluster-randomized trial comparing multispecialty services within the primary care setting to usual care. The intervention is an integrated, interdisciplinary program that guides all pain-related care for intervention patients. It is embedded into everyday clinical practice flow utilizing assessment measures and intervention staff directly from the clinical care system.	Study infrastructure built to support and bolster EMR-based patient reported outcome data collection.
Strategies and Opportunities to Stop Colon Cancer in Priority Populations (STOP CRC)	Does an evidence-based, culturally tailored approach increase colorectal cancer screening in clinics that serve minority and low-income populations?	Federally Qualified Health Center clinics	30,000 patients aged 50 to 74 with no evidence of having had a recent colorectal cancer screening exam (fecal test, sigmoidoscopy or colonoscopy), and no history of colorectal disease.	Cluster randomized trial comparing usual care to intervention. The intervention consists of an automated data-driven, electronic health record-linked program for mailing FIT kits (with linguistically appropriate pictographic instructions and return postage) to patients due for CRC screening.	STOP CRC allowed patient reminders to be sent by email or letter and used a standard, well-validated quality improvement process (Plan-Do-Study-Act cycles) to facilitate program adaptations.
Time to Reduce Mortality in End-Stage Renal Disease (TiME)	Does systematically implementing a hemodialysis session duration of at least 4.25 hours improve survival, reduce hospitalizations and improve quality of life for patients with end-stage kidney disease?	Two large dialysis provider organizations	6432 patients with end stage renal disease treated by thrice weekly maintenance hemodialysis	Cluster-randomized, parallel-group clinical trial for patients initiating treatment with maintenance hemodialysis. Facilities are randomized in a 1:1 distribution to either: Intervention arm: recommend dialysis session durations of at least 4.25 hours for all patients initiating hemodialysis treatment regardless of body size or dialysis solute clearance measurements, or Usual care arm: no trial-driven approach to session duration.	24 hour urine collection eliminated and the quality of life survey was changed to the survey administered as part of routine care.

### Participants/Raters

Raters were trial principal investigators (PIs) or other investigators from their team (*n* = 4), Coordinating Center staff (1), or NIH staff (6). Six of the raters had familiarity with all five trials either because they had participated in funding decisions or regular cross-project meetings. Two raters (both NIH staff) had limited knowledge of any of the projects prior to participating in the PRECIS 2 exercise. Raters were recruited based on their interest and availability. The six NIH staff rated all five trials. The PIs or other investigators, as well as the Coordinating Center staff, each rated only two trials, one of which was his/her own.

### Rating procedures

To measure the pragmatic nature of the NIH Collaboratory trials, we used the PRECIS-2 toolkit (https://crs.dundee.ac.uk/precis). The CONSORT workgroup on Pragmatic Trials created the PRECIS criteria to help trialists design trials that are pragmatic across multiple domains [1, 5]. While not primarily intended to analyze trials post hoc, the original PRECIS scale was successfully used for this purpose [5]. Based on findings from the initial use of the tool, a team at the University of Dundee developed the second version [6], which reduces the number of domains rated from 10 to nine, makes comparisons to usual care without explicit rating of the control conditions, and considers external validity in the recruitment and setting domains.

The PRECIS-2 toolkit includes nine domains: (1) eligibility - who is selected to participate in the trial; (2) recruitment - how participants are recruited into the trial; (3) setting - where the trial is being done; (4) organization - what expertise and resources are needed to deliver the intervention; (5) delivery flexibility - how the intervention is delivered; (6) adherence flexibility - what measures are in place to make sure participants adhere to the intervention; (7) follow-up - how closely participants are followed-up; (8) primary outcome - how relevant is it to participants; and (9) primary analysis - to what extent all data all included. Each domain is scored on a five-point Likert scale from very explanatory (1) to very pragmatic (5), with a score of 3 indicating that a trial is equally pragmatic and explanatory. In addition, an overall composite score is reported for a given trial to characterize the overall pragmatic nature of the trial. We also calculated an overall mean score for each domain across trials, to illustrate for which domains the trials, in general, were more or less pragmatic.

### Training

All raters received training in applying PRECIS-2. The training consisted of an orientation webinar by one author (RG) based on the PRECIS-2 toolkit, practice with a published protocol, and a second web conference to calibrate ratings by discussing ratings that differed among the individuals participating in the training. Following the training, each of the five demonstration projects was rated by eight raters and evaluated at two time points, using the initial grant application and a required progress report written at the end of a 1-year planning period that included changes to the protocol or implementation approach. Four trials included a rating by a trial team member. Each rater entered their two sets of ratings on a form, which included space for comments.

### Analysis

We produced one PRECIS wheel for each time point (pilot/planning phase and implementation phase) for each of the five trials using the PRECIS-2 Toolkit, which calculates the median scores for ratings for a given trial. The data were analyzed using STATA 12.0 (StataCorp, College Station, TX, US) and SAS 9.3. We calculated the mean and median scores and the range of scores for each domain, trial, and time point. We also calculated the differences in scores between the two time points for each of the PRECIS-2 domains for each trial. To evaluate change over time, we examined the spread in these differences and determined the level of statistical significance (for a given domain for a given trial) using the sign test in Stata 12.0, a Wilcoxon nonparametric test of equality of matched pairs. Interrater agreement or reliability was calculated for each trial using Gwet’s AC1 statistic [7], a more robust version [8] of Fleiss’ Kappa [9]. Interrater agreement was also measured using the intraclass correlation coefficient. Additionally, we obtained each trial principal investigator’s impressions on the degree of congruence of the ratings with on-the-ground experience.

To inform our secondary goal of assessing PRECIS-2 usability, we reviewed the comments provided by the raters for each trial. Two authors (KJ and GN) organized the comments by domain and indicated study design aspects that raters considered in their scoring that were not specified in the PRECIS-2 toolkit, as well as rating challenges. All raters reviewed these results.

## Results

### Comparison of domains across trials

All five demonstration projects were rated to be more pragmatic than explanatory. The overall composite scores, calculated on the basis of the average score of the means and medians across the domains, are all greater than 3 on a scale where 3 signifies equally pragmatic/explanatory. Mean and median scores for each trial at the implementation phase for each domain are presented in Table 2. Whereas all five trials were more pragmatic than explanatory (that is, overall composite median rating > 3.0), TiME and LIRE were found to be the most pragmatic (4.5 overall composite median rating for both). The domains for which those two trials were most pragmatic (that is, mean of eight raters > 4.5) were recruitment (mean of eight raters = 4.8) and follow-up (4.9) for LIRE, and eligibility (4.8), recruitment (4.6), follow-up (4.6), and primary outcome (4.9) for TiME. The domains for which they were less pragmatic but were still more pragmatic than explanatory (that is, mean of eight raters < 4.5 but > 3.0), were organization (3.8), setting (4.0), primary outcome (3.6), and eligibility (4.3) for LIRE and delivery (4.1), setting (4.4), organization (4.4), and adherence (4.4) for TiME. The trials that were less pragmatic but still more pragmatic than explanatory were ABATE and PPACT (overall composite median rating = 3.5 and 3.6, respectively.) The domains that were rated as more explanatory than pragmatic (that is, mean of eight raters < 3.0) were organization (2.6) and delivery (2.1) for ABATE and organization (2.9) and adherence (2.8) for PPACT. However, those two trials were also found to be very pragmatic (that is, mean of eight raters > 4) on several dimensions including recruitment (4.5) and analysis (4.5) for ABATE and primary outcome (4.6) and analysis (4.9) for PPACT. STOP CRC fell in between, with an overall composite median rating of 3.9. In general, the domains along which trials were, on average, most pragmatic included primary analysis (mean = 4.7 range = 4.5 to 4.9)), recruitment (4.3 (3.6 to 4.8)), eligibility (4.1 (3.4 to 4.8)), setting (4.1 (4.0 to 4.4)), follow-up (4.1 (3.4 to 4.9)), and primary outcome (4.1 (3.5 to 4.9)). On average, the less pragmatic, although still more pragmatic than explanatory, the domains were organization (3.3 (2.6 to 4.4)), flexibility of delivery (3.5 (2.1 to 4.5)), and flexibility of adherence (3.8 (2.8 to 4.5)).

**Table 2:** PRECIS scores by trial (at implementation phase) and domain

PRECIS domain	ABATE Infection	LIRE	PPACT	STOP CRC	TiME
	Mean (SD, Median)	Mean (SD, Median)	Mean (SD, Median)	Mean (SD, Median)	Mean (SD, Median)
Eligibility	3.9 (1.1, 4.0)	4.3 (0.9, 4.5)	3.4 (1.1, 3.5)	4.4 (0.5, 4.0)	4.8 (0.7, 5.0)
Recruitment	4.5 (1.1, 5.0)	4.8 (0.5, 5.0)	3.6 (0.9, 4.0)	3.9 (0.8, 4.0)	4.6 (0.7, 5.0)
Setting	4.0 (1.1, 4.0)	4.0 (0.9, 4.0)	4.0 (1.1, 4.0)	4.3 (0.7, 4.0)	4.4 (0.7, 4.5)
Organization	2.6 (1.2, 2.5)	3.8 (0.9, 3.5)	2.9 (0.8, 3.0)	2.9 (0.8, 3.0)	4.4 (1.1, 5.0)
Delivery	2.1 (1.0, 2.0)	4.5 (0.8, 5.0)	3.0 (0.5, 3.0)	3.8 (0.5, 4.0)	4.1 (0.6, 4.0)
Adherence	3.4 (1.4, 3.5)	4.5 (0.8, 5.0)	2.8 (0.7, 3.0)	3.8 (0.9, 3.5)	4.4 (0.7, 4.5)
Follow-up intensity	3.4 (1.2, 3.0)	4.9 (0.4, 5.0)	3.9 (0.6, 4.0)	3.9 (0.8, 4.0)	4.6 (0.5, 5.0)
Primary outcome	3.5 (1.1, 3.0)	3.6 (0.9, 4.0)	4.6 (0.5, 5.0)	3.9 (1.1, 4.0)	4.9 (0.4, 5.0)
Analysis	4.5 (0.8, 5.0)	4.5 (0.5, 4.5)	4.9 (0.4, 5.0)	4.9 (0.4, 5.0)	4.5 (0.8, 5.0)
Overall composite					
Median rating	3.5	4.5	3.6	3.9	4.5
Mean rating	3.5	4.3	3.7	4.0	4.5
AC1 statistic	0.08	0.29*	0.23*	0.28*	0.40*
Intraclass correlation coefficient	0.31	0.07	0.06	0.05	0.06

Note: Overall ratings are average scores

*p value ≤ 0.001

### Reliability

At the implementation phase, we found modest but statistically significant interrater agreement for four of the five trials (AC1 statistic ranged from 0.23 to 0.40, *p* values ≤ 0.001; Table 2). Intraclass correlation coefficients ranged from 0.05 to 0.31. Ratings for the planning phase were similar: AC1 statistics ranged from 0.11 to 0.44 and all *p* values were ≤ 0.001 (data not shown). We examined outliers to see if differences existed among raters who were and were not previously familiar with the studies or for raters who were rating their own project. We did not observe any notable patterns. However, the principal investigators’ on-the-ground experience did not always match the rater-assessed examples of change, as discussed further below.

### Change over time

**Fig. 1:**
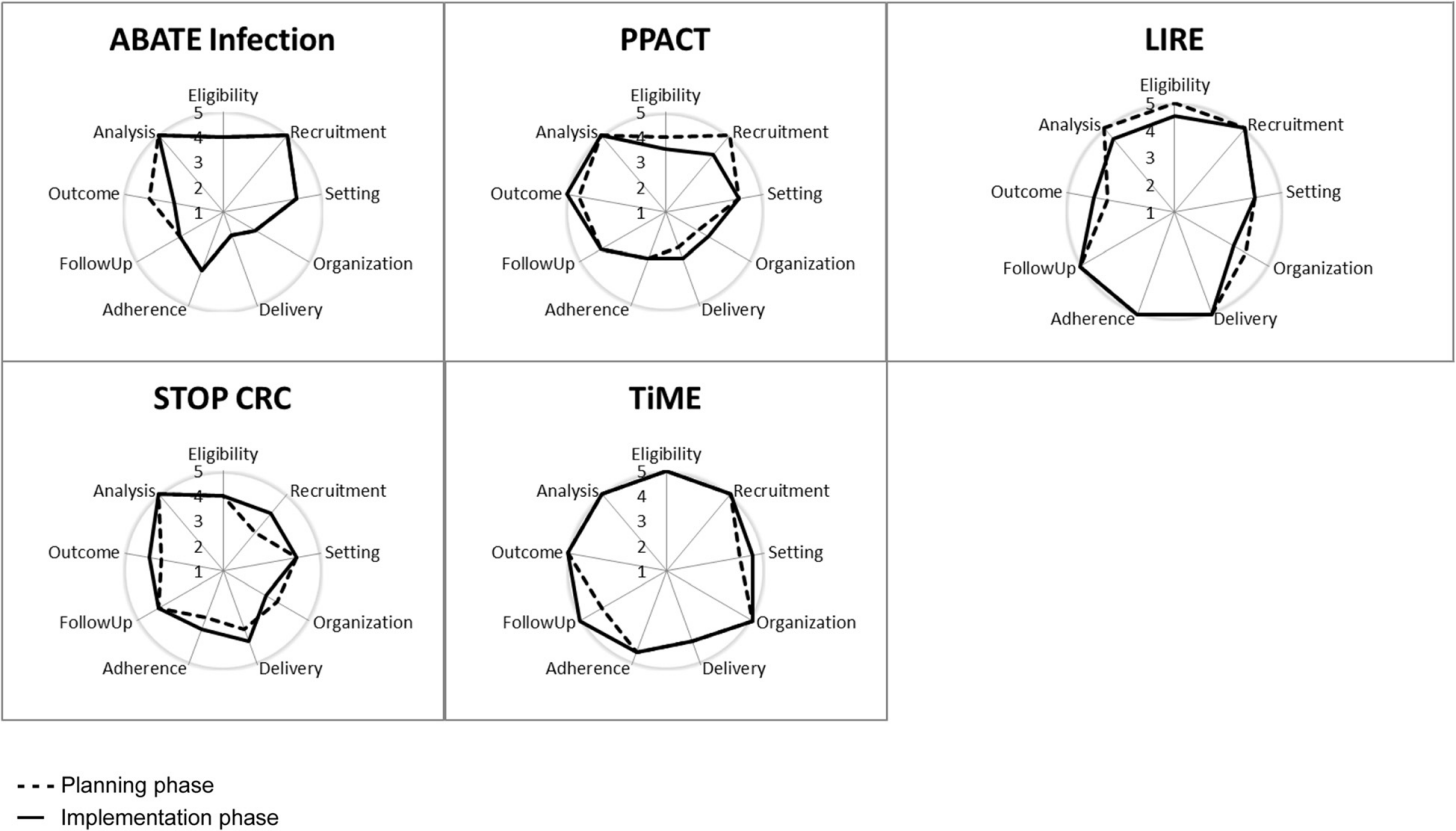
PRECIS wheels as assessed by raters for each of the five trials at two time points. Ratings on a 1 – 5 scale indicate more explanatory to more pragmatic ratings. The dashed line indicates the planning phase. The solid line indicates the implementation phase

Trial refinements over the course of the planning phase represented responses to logistical issues, stakeholder preferences, and input from the NIH Collaboratory members as the studies approached full implementation. Some examples are shown in Table 1. Figure 1 shows the PRECIS wheels for each of the five trials at the two time points. We found between one and three rater-assessed significant changes over time for each study; however, we did not note any consistency in terms of direction or domain. Additionally, the PIs agreed with the direction of the ratings for only one study. As an example, raters assessed that recruitment procedures became more pragmatic for one study, but the PI indicated that the story was more complex, with some aspects becoming more pragmatic but others less so. Study procedures were refined such that organizational approaches to patient prioritization did indeed more closely mirror everyday clinical care; however, the timing of patients receiving the intervention became more tied to study-specific provider randomization points.

### Rater comments

Table 3 summarizes factors that raters noted they had considered in rating the study. These were largely consistent with the examples in the PRECIS-2 toolkit that were included in the training materials. However, the raters’ comments about additional considerations that they factored into assigning domain ratings highlight that the PRECIS-2 ratings are not necessarily conclusive but generate a starting point for discussion, as we describe in more detail below.

**Table 3:** Examples of rater explanations for ratings of individual trials

Domain	More pragmatic: examples from training	More explanatory: examples from training	Study criteria noted by raters that training did not specifically address	Rating commentary
Eligibility: who is selected to participate in the trial?	Participants essentially identical to those who would receive the intervention if it was in usual care: both health care systems and patients	Lots of exclusions (for example, those who do not comply, respond to treatment, or are not at high risk for primary outcome, are children or elderly), or use many selection tests not used in usual care.	Cognitive impairment; clinic size; clinic willingness to participate; patient willingness to consent (more explanatory)	Differentiating site vs. patient eligibility
				Especially for complex or novel interventions, are exclusions based on comorbidities any different for intervention than what would occur in usual care?
Recruitment: how are participants recruited into the trial?	Usual appointment or clinic	Targeted invitation letters, advertising in newspapers, radio plus incentives and other routes that would not be used in usual care	Informed consent procedures; extra screening procedures in EHR (more explanatory)	None noted
Setting: where is the trial being done?	Setting of trial identical to usual care setting	A very explanatory approach would occur only a single center, or only specialized trial or academic centers	Diversity in terms of number, location, and organization type considered in very pragmatic ratings; regional studies, studies conducted in one type of health care system; consent requirements considered in more explanatory ratings	There are many dimensions to consider when comparing the setting to usual care. For example, was the goal to rate compared to a particular type of setting such as safety net clinics or to include multiple types of health systems? Additionally, participating institutions tended to have good quality improvement capacity; it is difficult to rate how this compares to “typical” settings.
Organization: what expertise and resources are needed to deliver the intervention?	Resources, provider expertise and the organization of care delivery in the intervention arm identical to usual care	Trial increases staff levels, gives additional training, requires more than usual experience or certification and increases resources	Space, intervention delivery, outreach (more explanatory). A train the trainer approach is one way to make training procedures more pragmatic.	It was noted that many of these trials take place in innovative health systems that may not be typical of the country, potentially conflating setting and organization.
				Difficult to determine weight or importance of organization at provider level versus organization at IT or administration level.
				The amount of effort undertaken to maintain ‘adherence’ at the organizational level was not particularly well documented.
Flexibility of delivery: how should the intervention be delivered?	Flexibility in delivering intervention identical to usual care	If there is a strict protocol, monitoring and measures to improve compliance, with specific advice on allowed co-interventions and complications	An example noted was that guidance in place but provider can deviate from it or the guidance can be modified over time based on stakeholder feedback	What happens if there is a strict protocol in usual care as well?
Flexibility of adherence: what measures are in place to make sure participants adhere to the intervention?	No more than usual encouragement to adhere to the intervention	Exclusion based on adherence, and measures to improve adherence if found wanting		Distinguishing adherence encouragement that would happen in usual care from the intervention. At the clinic/provider level, monitoring was interpreted to relate both to flexibility of delivery and adherence.
Follow-up: how closely are the participants followed-up?	No more than usual follow-up	More frequent, longer visits, unscheduled visits triggered by primary outcome event or intervening event, and more extensive data collection	Use of electronic records (more pragmatic); additional contacts, additional measures (more explanatory)	None noted
Primary outcome: how relevant is it to participants?	Outcome measure very relevant to participants	Using a surrogate, physiological outcome, central adjudication or use assessment expertise that is not available in usual care, or the outcome is measured at an earlier time than in usual care	Implementation feasibility or other procedural details (more explanatory)	How to handle clinic- versus patient-relevant outcomes
Primary analysis: to what extent are all data included?	intention to treat with all available data	Excludes ineligible post-randomization participants, includes only completers or those following the treatment protocol		Extensive analytic details were not available in the materials raters had available.

Raters noted several challenges in applying the criteria in their commentary. For the *eligibility* domain, raters had to consider both eligibility of the facility and eligibility of the patient. For example, for most of the trials, participating facilities or clinics were selected by convenience and no information was available about inclusion criteria even though this was the unit of randomization. Facility willingness to participate was viewed by raters as an aspect of eligibility that was more explanatory than pragmatic.

The *setting* and *organization* domains proved particularly difficult to rate relative to usual care. Raters commented that there are many different aspects to consider when rating settings within the diverse U.S. health system, including geography, types of care provided, and financing (for example, fee for service versus managed care). Furthermore, institutions where these trials were occurring all had the resources and infrastructure to support a systems-change intervention, making it possible that these institutions had relatively high organizational resources to support complex quality improvement. Even if this infrastructure was not research-specific, this potential difference from usual care led to more explanatory ratings of the setting and organization domains.

For the *flexibility of delivery* domain, the determination of whether an intervention was relatively more restrictive than a strict quality control protocol in usual care was challenging. Similarly, it was challenging to rate the *flexibility of adherence* for an intervention relative to usual care because, if the intervention was successful, the adherence procedures could become usual care. Second, few studies documented extensively efforts undertaken to maintain “adherence” at the organizational level. For example, when leadership changes occur, the need arises for substantial discussion and planning to continually “engage” the stakeholders/leadership in the health system. Most of these activities are not planned but are undertaken ad hoc when health systems lose their leadership. To what extent these efforts to re-engage leadership in the conduct of the trial represented less pragmatic adherence is unclear.

*Primary outcome* is rated according to the extent to which it is relevant to participants, but raters struggled with how to rate outcomes that might be more important to health systems than to patients, for example, process efficiency. Raters also had to determine how to factor in criteria that pertained to multiple domains, and whether they should “be counted” more than once. For example, consent by patients or organizational willingness to participate pertained to multiple domains: eligibility, recruitment, setting. Thus, some raters attempted to provide an average score across unrelated subdomains; other raters may have only considered a single subdomain.

## Discussion

The objective of this study was to analyze five pragmatic trials in order to characterize pragmatic versus explanatory design by PRECIS-2 domains and how design details changed over the course of a yearlong study-planning period. In five trials designed as pragmatic trials in diverse U.S. healthcare settings, we observed that trials were designed as more pragmatic than explanatory as measured by all PRECIS-2 domains.

Raters struggled to use the PRECIS system for this analysis, as illustrated by the comments in Table 3, PI discussions, and the ICC range from 0.05 to 0.31. For comparison, in a study by Glasgow et al., in which they studied three effectiveness trials of weight loss in obese patients with comorbid conditions [5], the ICC for individual items was 0.72, and the overall kappa interrater reliability on the composite PRECIS score was *r* = 0.88. The large difference in interrater reliability is surprising given that we used a similar training approach and had access to detailed study information.

Whereas the rating challenges limit our ability to draw conclusions about specific studies, some general observations emerge. Across studies and time points, the domains rated as most pragmatic were analysis and recruitment, whereas those that were closer to explanatory (average range 3 to 4) were organization, delivery, and adherence. This could reflect, in part, that it may be easier to be pragmatic for some domains than for others. For example, it is relatively easy to be pragmatic for patient eligibility by taking all comers; but it is often difficult to be pragmatic when trying to deliver an intervention.

It is important to note that explanatory elements of pragmatic design do not necessarily relate to study quality. Some trial aspects may need to be designed in a more explanatory manner in order to answer the study question. PRECIS-2 ratings provide guidance to researchers on the appropriate corresponding study procedures. For example, the more explanatory rating of the organization domain (how the resources, provider expertise and the organization of care delivery in the intervention arm of the trial compare to usual care) indicates that the study involves extra resources such as training. By noting this during the design phase, study teams can make sure they communicate with involved health systems about time and resource requirements. However, it is important to note that requiring additional training does not necessarily make the organizational domain more explanatory: the same approaches to training personnel to roll out a trial intervention could be the same as those approaches used by the organization to roll out a clinical or quality improvement intervention.

It is difficult to compare our overall findings to other reports that rated studies using PRECIS domains because they utilized an earlier version of the tool and had different study questions. However, one other study has used PRECIS criteria to examine change over time. Elder and Munk [10] used a modified PRECIS wheel to obtain input on study methodology while planning a new phase of research examining two complementary therapies for chronic low back pain. The study led to re-evaluation of the design of certain aspects, for example participant characteristics, that were rated as more explanatory than expected and could be made less restrictive. However, as in our experience, the authors concluded that having a more explanatory characteristic within a pragmatic trial may be appropriate depending on the research question.

This study generated insights that may be useful for future use or refinement of the PRECIS-2 tool. As per Table 3, raters struggled with how to apply ratings. In particular, comparing the intervention to usual care requires guidance about 1) how to handle domains such as recruitment that can pertain to health care settings or patients; 2) how to rate a systems-change intervention (which could become, but is not currently, standard care) and 3) what level of existing supports/standards, for example communication with leadership or use of electronic health record functionality, is considered typical in usual care. After this study was completed, the PRECIS-2 designers published a manuscript that fully described the PRECIS-2 tool [3]. Their explanation of the domains in detail highlights that the complexities we encountered resonated with theirs, including how to rate an intervention that is designed to change usual care and how local care nuances (for example, data systems) can influence ratings. The challenge of using the tool, especially for some criteria, suggests that the PRECIS-2 criteria may need to be further refined in order to have sufficient specificity to enable comparison of intervention to usual care in the context of a broad range of settings. The issue is not just tool development, but also clarification of how we characterize care and what components are “usual”. Having this more detailed understanding of usual care and guidance regarding how to characterize care would enable a more clear understanding of the degree to which the intervention differs from usual care and has practical utility to a health system, given the diversity of health systems that exist.

In addition, it would be helpful to have guidance about the amount of study information that teams need in order to best use the PRECIS-2 tool. If we had this guidance, the benefit would be that all trials could more easily capture information. In turn, this would enable better comparisons across trials and allow for analysis of a broader trial portfolio. Additionally, looking across trials and across time points would be useful, but we were limited by what information we had. Therefore, providing guidance on what information is needed to best apply this tool would better enable its utility.

The materials that we used to rate the study were the grant application and progress report, which contained many details that pertained to the PRECIS-2 domains but were not organized according to PRECIS-2 domains. The information in these documents may have contributed to low interrater reliability; however, they contained a substantial amount of implementation-oriented information. It is possible that ratings would have been even more difficult using the details typically available in a protocol or manuscript of study findings. It also is possible that the limited number of raters for each study could have contributed to the low interrater reliability. However, in both the Loudon et al. paper [3] and our experience, ratings ultimately benefited from local familiarity with the health system where the trial was being conducted and direct input from study team members helped prompt discussion and clarification about study details. As such, we do not necessarily see value in review by an external group, except for providing advice about how to best use the PRECIS-2 tool. The ability of the PRECIS-2 framework to support discussions about how to interpret and operationalize design decisions is helpful.

## Conclusions

The raters participating in the process found it an informative way to learn about pragmatic design in general as well as about specific studies. This study demonstrates that PRECIS-2 can be used to rate protocols, as well as for study planning, and helps address the need for systematic approaches to reporting pragmatic studies [11]. However, results of analyses using the criteria post hoc should factor in the challenges encountered in our analysis. In addition, refinements would be helpful for raters. These could include creating additional rating criteria, linked to numerical rating, and exploring different formats for brief training of raters. Our results highlight that researchers should anticipate making changes to study protocols for pragmatic trials based on health system realities.

## Abbreviations

LIRE: A Pragmatic Trial of Lumbar Image Reporting with Epidemiology
NIH: National Institutes of Health
NIH Collaboratory: National Institutes of Health Healthcare Systems Research Collaboratory
PI: Principal Investigator
PRECIS-2: Pragmatic Explanatory Continuum Indicator Summary framework, 2nd version
TiME: Time to Reduce Mortality in End-Stage Renal Disease trial
